# QTL mapping and epistatic interaction analysis in asparagus bean for several characterized and novel horticulturally important traits

**DOI:** 10.1186/1471-2156-14-4

**Published:** 2013-02-02

**Authors:** Pei Xu, Xiaohua Wu, Baogen Wang, Tingting Hu, Zhongfu Lu, Yonghua Liu, Dehui Qin, Sha Wang, Guojing Li

**Affiliations:** 1 Institute of Vegetables, Zhejiang Academy of Agricultural Sciences, 310021, Hangzhou, People’s Republic of China

**Keywords:** Asparagus bean, Cowpea, Epistasis, Flowering time, Leaf senescence, Node to first flower, Pod number, QTL, RIL

## Abstract

**Background:**

Asparagus bean (*Vigna. unguiculata*. ssp *sesquipedalis*) is a subspecies and special vegetable type of cowpea (*Vigna. unguiculata* L. Walp.) important in Asia. Genetic basis of horticulturally important traits of asparagus bean is still poorly understood, hindering the utilization of targeted, DNA marker-assisted breeding in this crop. Here we report the identification of quantitative trait loci (QTLs) and epistatic interactions for four horticultural traits, namely, days to first flowering (FLD), nodes to first flower (NFF), leaf senescence (LS) and pod number per plant (PN) using a recombinant inbred line (RIL) population of asparagus bean.

**Results:**

A similar genetic mode of one major QTL plus a few minor QTLs was found to dominate each of the four traits, with the number of QTLs for individual traits ranging from three to four. These QTLs were distributed on 7 of the 11 chromosomes. Major QTLs for FLD, NFF and LS were co-localized on LG 11, indicative of tight linkage. Genome wide epistasis analysis detected two and one interactive locus pairs that significantly affect FLD and LS, respectively, and the epistatic QTLs for FLD appeared to work in different ways. Synteny based comparison of QTL locations revealed conservation of chromosome regions controlling these traits in related legume crops.

**Conclusion:**

Major, minor, and epistatic QTLs were found to contribute to the inheritance of the FLD, NFF, LS, and PN. Positions of many of these QTLs are conserved among closely related legume species, indicating common mechanisms they share. To our best knowledge, this is the first QTL mapping report using an asparagus bean × asparagus bean intervarietal population and provides marker-trait associations for marker-assisted approaches to selection.

## Background

Cowpea (*V. unguiculata* L. Walp.) is an important grain legume, fodder and vegetable crop in many tropical and subtropical regions of the world [1,2]. Asparagus bean (*V. unguiculata* ssp. *sesquipedialis*), also known as ‘yard long’ bean or snap bean is a subspecies and distinctively domesticated type of cowpea grown mainly for vegetable use in many Asian countries. Asparagus bean, together with the African cowpea (*V. unguiculata* ssp. *unguiculata*), forms the two main divisions of cultivated cowpea [3]. Due to selection towards traits favorable for vegetable use, the present day asparagus bean differs a lot from African cowpea in many aspects including plant architecture (climbing versus erect), growth habit (indeterminate versus determinate), pod length and pod fiber content [4,5].

Recent progress in cowpea genomics has provided an opportunity to unravel the genetic basis of horticulturally important traits in asparagus bean. Bead-assay SNP genotyping was recently used to build a consensus genetic map which includes more than 1,000 loci from as many as thirteen different RIL populations [6,7]. Among the 13 mapping populations, one is derived from an inter-varietal asparagus bean cross. This population has also been used to develop a separate, but comparable, genetic linkage map of asparagus bean by integrating many SSR markers [8]. This subspecies/population-specific map has been especially useful in mapping two qualitative traits, namely, flower and seed coat color in the authors’ lab [9].

For commercial asparagus bean production, tender and crisp immature pods during early development is desired. This can be, at least in part, accomplished by breeding early-flowering varieties with the potential to yield more pods per plant. Early flowering is known to be related to node position at which first inflorescence occurs; therefore, the node to first flower has been practically used as an indirect indicator of earliness [10]. Another desirable trait of asparagus bean is extended longevity or delayed plant senescence. This characteristic usually allows for two or more flushes of flowering, potentially resulting in more pods per plant. The aforementioned traits including days to first flowering (FLD), nodes to first flower (NFF), leaf senescence (LS) and pod number per plant (PN) are among the most horticulturally important traits in asparagus bean. All four traits are inherited quantitatively based on their field behaviors, and as such, dissecting their genetic basis calls for adequate statistical methods such as bi-parental QTL mapping.

Thus far, QTL mapping has not been reported in asparagus bean × asparagus bean populations; however, chromosome regions associated with horticultural/domestication-related traits have been mapped using cowpea populations of ssp. *unguiculata* pedigree. QTLs for 24 domestication-related traits were mapped using two temporal segregation populations derived from an asparagus bean × wild cowpea cross [11]. QTLs for seed weight and pod shattering were mapped using a normal cowpea × wild cowpea RIL population [12]. Earlier works include those which focused on a wide range of horticultural traits including organ sizes, yield components, plant height etc. [13-15]. Unfortunately these works remained anchored to the marker technology in which they were discovered. Here we present the identification of QTLs for four horticulturally important traits using an asparagus bean intervarietal RIL population. Two of these traits i.e. FLD and PN have been investigated previously using different plant materials [11] while the other two (LS and NFF) have been not. Many of the marker-trait associations we report are accessible via community genotypipng platforms and are useful for modern breeding, comparative genomics, and map-based cloning.

## Results

### Phenotypic analysis

**Figure 1:**
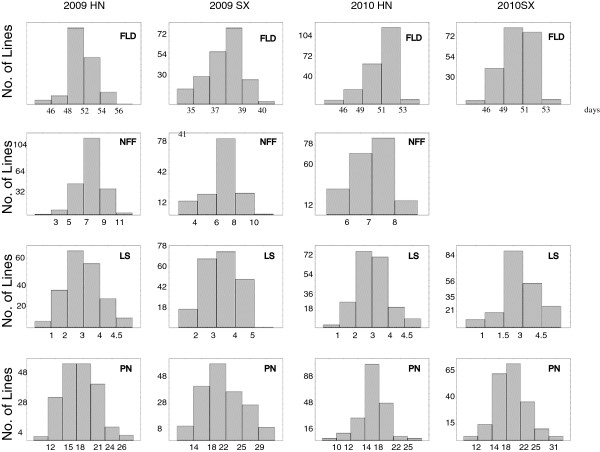
**Distributions of the phenotypic data in the ZN016 × ZJ282 RIL population.** FLD, days to first flowering; NFF, nodes to first flower; PN, pod numbers per plant; LS, leaf senescence.

In all experiments, the female parent ZN016 initiated flowering later than the male parent ZJ282, with the position of nodes to first flower being higher on the main stem. After flowering, ZN016 displayed a delayed senescence phenotype compared with ZJ282 and as a result produced greater number of pods per plant throughout its longer lifespan (Table 1). All four traits displayed a continuous distribution in the RIL population (Figure 1), with the population means falling between the parental values. Transgressive segregation was observed for FLD and PN as data of some offspring lines distributed beyond the parental values (Table 1), suggesting the existence of intragenic or intergenic interactions. Generally low FLD values were observed in SX2009, because of the late sowing of seeds in a warmer season (see ‘methods’ below). An underestimate of PN in both experiments of year 2010 was noticed, which was due to a long rainy weather that caused pod dropping; however, this didn’t affect detection of QTLs with major effects (see below). Results of ANOVA showed that the between-line variations of all four traits in each trial were significant at p=0.0001 except for PN_SX2010, which was significant at p=0.001. The broad-sense heritability of the four traits ranged from 35% (PN) to 60.3% (FLD). The heritability of LS and NFF was 57.5% and 56.7%, respective. The strikingly low heritability of PN was believed due to the effect of pod dropping in 2010, as a separated estimate of PN heritability for each year turned out to be over 60% (data not shown).

**Table 1:** Mean and range of the traits in the parents and the RIL population

**Location**	**Trait**			**2009**				**2010**	
		**P1**^**a**^	**P2**	**RILs**		**P1**	**P2**	**RILs**	
				**Mean**	**Range**			**Mean**	**Range**
HN^b^	FLD^c^	53.5	44.5	51.5	44.3–55.3	52.3	43	50.1	45–54.3
	NFF	9	4	8	3–12	8	2	6.9	3–9
	LS	0	5	3.0	0.5–5	0.5	5	3.1	0.8–5
	PN	30.3	16.4	18.7	10–31.4	19.3	10.8	16.6	8.9–27.3
SX	FLD	44.5	36.5	39	33–42	53	46	50.6	45–54
	NFF	9	4	7.1	3–11	NA^d^	NA	NA	NA
	LS	2	5	3.5	1–5	0	5	3.0	0.5–5
	PN	35.5	16.5	21.3	10.8–33.8	20.3	19.3	18.6	9.0–33

^a^ P1: ZN016 (♀), P2: ZJ282 (♂), RILs: recombinant inbred lines.

^b^ HN: Haining, SX: Shaoxing.

^c^ FLD: days to first flowering, NFF: nodes to first flower, LS: leaf senescence, PN: pod number per plant.

^d^ NA: data of LS not available for this trial.

Trial-wide correlation analyses of individual traits revealed high correlation coefficients (r≥0.55) for FLD and LS at a significant level of p=0.0001 (Table 2). Correlations were also highly significant (p=0.0001) for NFF among different trails, with the coefficients ranging from 0.36 to 0.46. Correlations of PN between different years in the same location were weak, but those between locations in the same year were considerably strong as shown by the correlation coefficients of 0.36 and 0.48 for the two years, respectively. The long rainy weather in 2010 as mentioned above was considered the main reason for phenotypic inconsistencies of PN between years. Across traits, significant positive correlations were observed between FLD and NFF (mean r=0.50, P<0.0001), whereas FLD and LS were negatively correlated (mean r=−0.39, P<0.0001). Significant negative correlation was found between NFF and LS in 4 and 6 out of the 16 environment combinations at P=0.0001 and P=0.01 significance levels, respectively (Additional file 1).

**Table 2:** The correlation coefficients of individual traits among different trials

**Trait**	**Trail**	**2009HN**^**a**^	**2009SX**	**2010HN**	**2010SX**
FLD^b^	2009HN	1			
	2009SX	0.671^**^	1		
	2010HN	0.708^**^	0.645^**^	1	
	2010SX	0.653^**^	0.582^**^	0.709^**^	1
NFF	2009HN	1			
	2009SX	0.463^**^	1		
	2010HN	0.380^**^	0.355^**^	1	-
LS	2009HN	1			
	2009SX	0.630^**^	1		
	2010HN	0.656^**^	0.550^**^	1	
	2010SX	0.605^**^	0.548^**^	0.591^**^	1
PN	2009HN	1			
	2009SX	0.36^*^	1		
	2010HN	0.27^**^	0.231	1	
	2010SX	0.191	0.176	0.483^**^	1

^a^ HN: Haining, SX: Shaoxing.

^b^ FLD: days to first flowering, NFF: nodes to first flower, LS: leaf senescence, PN: pod number per plant.

^**^Significant at P≤0.0001, ^*^significant at P≤0.01.

### QTL analysis

#### Days to first flowering (FLD)

**Figure 2:**
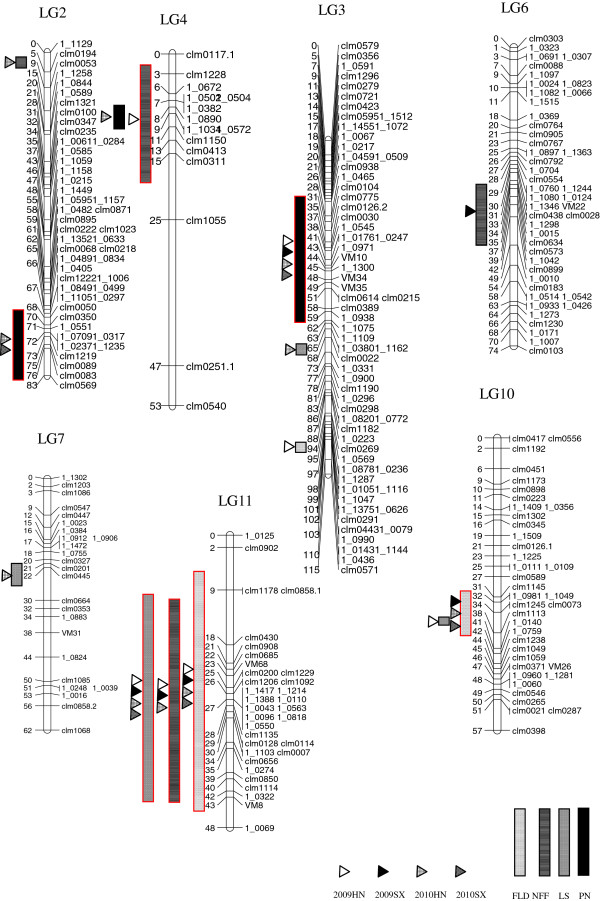
**Locations and intervals of QTLs for each of the four traits detected in the current study.** Intervals of QTLs were indicated by rectangles, and the trials in which the QTLs were detected were marked by triangles. QTLs with LOD scores above 3 were shown in red.

A major QTL, *Qfld.zaas-11,* was detected on LG11 in all environments with the LOD scores ranging from 5.3 to 10.6 (Table 3). It explained up to 31.9% of phenotypic variance. The interval length of *Qfld.zaas-11* ranged from 26.8 cM to 36.9 cM in the four trials, with the region from position 10.3 cM to 37.1 cM being consistently covered in multiple trials (Table 3, Figure 2). The ZJ282 allele of *Qfld.zaas-11* advanced flowering by 2.2 days in the four trials and was tagged with the closest marker 1_0043. Another QTL, *Qfld.zaas-10*, was detected on LG10 twice with the LOD scores higher than 3. It explained 16% of the phenotypic variance on average. The early-flowering allele of *Qfld.zaas-*10 was also donated by ZJ282. In the HN2009 experiment, the substitution of the ZN016 allele with the ZJ282 allele of *Qfld.zaas-10* advanced flowering by 1.9 days when estimated with the nearest locus *Clm1113*.

**Table 3:** QTLs detected with a LOD score higher than 3 in the four trials

**QTL**	**Location/LG**	**Methods of QTL detection**	**Position (cM)**	**Length (cM)**	**Closest marker**	**MQM LOD**^**c**^	**MQM R**^**2**^**(%)**	**Additive effect**
	**HN2009**							
*Qfld.zaas-10*^*a*^	LG10	IM, MQM^b^	28.7-50.1	21.4	Clm1113	3.94	17.2	0.89
*Qfld.zaas-11*	LG11	IM, MQM	10.3-37.1	26.8	1_1103	10.59	40.1	1.35
*Qnff.zaas-4*	LG4	MQM	2-19.4	17.4	1_0504	3.23	9.9	0.48
*Qnff.zaas-11*	LG11	IM, MQM	10.3-42.3	32	Clm1206	8.44	33.3	0.86
*Qpn.zaas-3*	LG3	IM, MQM	47.3-61.7	14.4	Clm0614	4.53	20.1	1.7
*Qls.zaas-11*	LG11	IM, MQM	9.3-42.3	33	1_1103	11.2	42.1	−0.69
	**SX2009**							
*Qfld.zaas-10*	LG10	IM, MQM	31.5-39.7	8.2	VM26	5.6	23.6	0.9
*Qfld.zaas-11*	LG11	IM, MQM	10.3-42.3	32	1_0043	5.32	22.7	0.89
*Qls.zaas-11*	LG11	IM, MQM	22.6-36.1	13.5	1_1103	3.51	16.1	−0.32
	**HN2010**							
*Qfld.zaas-11*	LG11	IM, MQM	6.4-42.3	35.9	1_0043	10.54	40.1	1.1
*Qnff.zaas-11*	LG11	IM, MQM	13.3-41.5	28.2	Clm1135	4.98	22.5	0.61
*Qpn.zaas-3*	LG3	IM, MQM	49.3-56.6	7.3	Clm0614	5.11	21.9	1.62
*Qls.zaas-11*	LG11	IM, MQM	11.3-42.3	31	Clm1135	6.98	29	−0.5
	**SX2010**^**c**^							
*Qfld.zaas-11*	LG11	IM, MQM	6.4-43.3	36.9	1_0043	5.91	24.8	0.91
*Qpn.zaas-2*	LG2	IM, MQM	69.4-79.1	9.7	Clm0089	3.56	16.1	2.31
*Qpn.zaas-3*	LG3	IM, MQM	29.2-61.7	32.5	Clm0614	6.11	26.1	2.17
*Qls.zaas-11*	LG11	IM, MQM	10.3-42.3	32	Clm0007	6.06	26.7	−0.58

^a^ FLD: days to first flowering, NFF: nodes to first flower, LS: leaf senescence, PN: pod number per plant.

^b^ IM: interval mapping, MQM: multiple-QTL mode mapping.

^c^ The trait of NFF not investigated in this experiment.

A plausible minor QTL was found on LG3 in SX2010 by both interval mapping (IM) and multiple-mode QTL mapping (MQM) (Table 4, Figure 2). It passed only the permutated LOD threshold and explained ~10% of the phenotypic variation.

**Table 4:** QTLs with a LOD score passing the permutated threshold but lower than 3

**QTL**	**Location/LG**	**Methods of QTL detection**	**Position (cM)**	**Length (cM)**	**Closest marker**	**MQM LOD**^**c**^	**LOD threshold**	**MQM** **R**^**2**^**(%)**	**Additive effect**
	**SX2009**								
*Qnff.zaas-6*	LG6	IM, MQM	28.3-35.4	7.1	Clm0438	2.96	1.9	13.2	0.83
*Qnff.zaas-11*	LG11	IM, MQM	30-35.1	5.1	1_1103	2.29	1.8	10.6	0.73
*Qpn.zaas-3*	LG3	IM, MQM	50.3-52.6	2.3	Clm0614	2.32	2.2	12.1	1.85
	**HN2010**								
*Qfld.zaas-10*^*a*^	LG10	IM, MQM^b^	31.5-38.7	7.2	1_0981	2.4	2	11.4	0.59
*Qnff.zaas-2*	LG2	IM, MQM	8.8-12.3	3.5	Clm0053	2.28	2.2	11.7	0.44
*Qpn.zaas-4*	LG4	IM, MQM	7.7-9.0	1.3	1_1034	2.43	1.8	11.3	1.19
*Qls.zaas-3*	LG3	IM, MQM	64-67	3	1_0380	2.43	2	11.4	−0.32
*Qls.zaas-7*	LG7	IM, MQM	20.7-25.5	4.8	Clm0445	2.46	1.9	11.4	−0.32
	**SX2010**^**c**^								
*Qfld.zaas-3*	LG3	IM, MQM	93-94.1	1.1	Clm0269	2.33	2.3	10.7	0.6
*Qfld.zaas-10*	LG10	IM, MQM	31.5-42.6	11.1	1_0140	2.61	2	11.8	0.62

^a^ FLD: days to first flowering, NFF: nodes to first flower, LS: leaf senescence, PN: pod number per plant.

^b^ IM: interval mapping, MQM: multiple-QTL mode mapping.

^c^ The trait of NFF not investigated in this experiment.

Only QTLs detectable by both IM and MQM were included.

#### Nodes to first flower (NFF)

NFF was scored in all trials but SX2010. Only one major QTL, *Qnff.zaas-11,* was detected in all three experiments, with the average LOD score being 4.6. This QTL explained as much as 22.1% of the phenotypic variation on average. Across experiments, the QTL peak positions only slightly shifted (0.8-1.6 cM apart). Estimated with the closest locus *1_1103*, the ZJ282 allele of this QTL lowered the number of node to first flower by 21% in the three trials. Apart from *Qnff.zaas-11*, one more QTL, *Qnff.zaas-4*, was detected on LG4 with a LOD score as high as 3.23 in HN2009 (Table 3, Figure 2).

Another two QTLs detectable under both IM and MQM models were found on LG2 and 6, respectively. Their LOD scores varied from 2.28 to 2.96 and explained 11.7% to 13.2% of the phenotypic variation individually, therefore were considered plausible minor QTLs (Table 4, Figure 2).

#### Pod number per plant (PN)

A major QTL, *Qpn.zaas-3*, was consistently detected on LG3 with an average LOD score of 4.5 (Table 3, Figure 2). This QTL could account for 20.1% of the phenotypic variation on average. The peak of *Qpn.zaas-3* coincided with the SSR locus *Clm0614*, with which the ZN016 allele of *Qpn.zaas-3* was estimated to contribute 1.6 more pods per plant (7.9% increment) in the four trials. On LG2, another QTL, *Qpn.zaas-2,* was detected in SX2010 (LOD=3.56). This QTL was also detectable in HN2010 but with lower LOD score (2.28). The DNA markers closest to the QTL peaks were only 4.1 cM apart between the two locations. *Qpn.zaas-2* had a moderate effect on PN, as revealed by the average phenotypic variation (16.1%) it explained. The *Qpn.zaas-2* allele with a positive effect on pod number was also carried by ZN016.

Passing the permutated LOD threshold and detectable by both IM and MQM, *Qpn.zaas-4* on LG4 accounted for 11.3% of the phenotypic variation and was considerd a plausible minor QTL for PN (Table 4, Figure 2).

#### Leaf senescence (LS)

A major QTL *Qls.zaas-11* explaining 28.5% of the phenotypic variation on average was detected with high LOD score in each of the four trials (Table 3, Figure 2). Across trials, the markers closest to QTL peak were less than 2 cM apart. Estimated with the closest locus *1_1103* in two of the four trials, the female allele of *Qls.zaas-11* conferred a delayed senescence phenotype by reducing the average senescence index from 3.6 to 2.7.

Two minor QTLs were detected in the HN2010 experiment to be localized on LG3 and LG7, respectively. They each explained ~11% of the phenotypic variation (Table 4, Figure 2).

### Genome wide epistasis interaction

Three and only one pair of loci showing significant epistatic interactions were detected in at least two of the four trials for the traits FLD and LS, respectively (Table 4). In each of the three two-locus interactions for FLD, one locus (*Clm0114*) was in the *Qfld.zaas-11* interval (2.2 cM apart to the peak), while the other was not independently related to FLD. For LS, the interactive pair of loci detected included a locus (*Clm1135*) in the peak region of the major QTL *Qls.zaas-11* and an independent locus on LG 8.

The chromosome region harboring *1_0514/1_0542* on LG 6 interacted differently from that harboring *Clm0364* on LG 5 with *QFld.zaas-11*. In the presence of the ZJ282 allele of *QFld.zaas-11*, the co-existence of the ZN016 allele of *1_0514/1_0542* shortened FLD significantly (P = 0.01) than of the corresponding ZJ282 allele (Table 5). In contrast, the combination of the ZJ282 alleles of both *QFld.zaas-11* and Clm0364 caused earlier flowering, relative to the ZJ282 allele of *QFld.zaas-11* combined with Clm0364 allele from ZN016. For LS, the ZJ282 allele of Clm0549 showed synergetic effect with the *Qls.zaas-11* allele from the same parent to cause more severe senescence.

**Table 5:** Two-locus interactions identified in at least two trials

**Trait**	**Locus1/**	**Locus2/**	**Trial**	**LRS**^**a**^			**Mean phenotype ± SE**^**c**^			
	**LG or QTL1**	**LG or QTL2**		**Interaction**	**Total**	**THR**^**b**^	***AABB***	***AAbb***	***aaBB***	***aabb***
FLD^d^	*1_0514/*	*Clm0114/*	2009HN	8.6	44	43.9	52.3	48.3	52.1	50.7
	*LG6*	*Qfld.zaas-11*	2010HN	10.8	56.9	42.3	±0.2	±0.3	±0.2	±0.3
	*1_0542/*	*Clm0114/*	2009HN	8.6	44	43.9	52.3	48.3	52.1	50.7
	*LG6*	*Qfld.zaas-11*	2010HN	10.8	56.9	42.3	±0.2	±0.3	±0.2	±0.3
	*Clm0364/*	*Clm0114/*	2009HN	6.7	51.7	43.9	52.3	50.6	52.2	48.7
	*LG5*	*Qfld.zaas-11*	2010HN	7.7	59.4	42.3	±0.2	±0.2	±0.2	±0.4
LS	*Clm0549/*	*Clm1135/*	2010HN	9.8	46.9	35.5	2.7	3.2	2.3	4.2
	*LG8*	*Qls.zaas-11*	2010SX	11.5	38.8	37.3	±0.2	±0.2	±0.2	±0.1

^a^ LRS: likelihood ratio statistic.

^b^ THR: threshold of total LRS.

^c^ Capital letter, ZN016 allele; lower case letter, ZJ282 allele.

^d^ FLD: days to first flowering, LS: leaf senescence.

## Discussion

### Modes of genetic control on the four traits in the ‘ZZ’ population

In the current study, three to four QTLs were identified for each of the four traits, covering 7 of the 11 asparagus bean chromosomes. Although some of the minor QTLs, in particular those being environment-dependent, still need verification, an apparent common feature of the genetic control of these traits is that they all comprise only one major QTL plus a few minor QTLs. Genetic modes of FLD and PN have also recently been dissected in an asparagus bean × wild normal cowpea population, in which a similar genetic pattern was disclosed [11]. NFF and LS were not investigated in study [11], but in the IT84S-2049 × 524B cowpea population, a single major QTL for NFF that explained up to 21% of the phenotypic variation was mapped despite no minor QTL was detected under the given statistic criteria [14]. These results suggested that alleles for these traits were selected similarly in differentiated genetic backgrounds. We suppose that mutations of major effect genes followed by selection and fixation is not only the main force causing cowpea/asparagus bean divergence but also the impetus shaping asparagus bean intervarietal variations. An interesting future task is to compare the patterns of mutations among the causal genes of these QTLs, both intervarietally and at the inter-subspecies level.

Additive effects clearly serve as the major genetic basis of the four traits while epistasis is also important for FLD and LS. Epistatic control of traits in cowpea/ asparagus bean has not been previously reported. A common feature of the three epistatic interactions detected is that they all occur between QTLs with main additive effects (MepQTLs) and QTLs showing epistatic effects only (epQTLs). Interestingly, we found the epistatic interaction could be in either coupling phase or in repulsion phase, depending on the locus, suggesting that some loci are co-adapted during domestication/speciation and that loci favorable for breeding purpose may exist in both improved cultivars and landraces. In other plant species including, soybean [16,17], oilseed brassica [18] and wheat [19], epistatic control of flowering time and leaf senescence has been reported. In the near future, it is required to validate and fine-dissect the epistatic QTLs under more uniform environments such as greenhouses.

### Co-localization of major QTLs governing FLD, NFF and LS

Significant correlations were found among FLD, NFF and LS, which are indicative of related or pleiotropic genetic factors governing these traits. This was then verified by the co-localization of major QTLs for these traits on LG11. Given that the *Qfld.zaas-11* and *Qls.zaas-11* alleles from either parent had accordant effects (advance flowering meanwhile promote senescence, or vice versa), this may also explain why almost all early flowering progenies were prematurely senesced. In two inter-subspecies crosses, QTLs for the traits of organ sizes (pod, seed and leaf) [11], as well as days to flowering and 100-seed weight [15], were also co-located. In garden pea, a major locus (*Lf*) for flowering time was reported to coincide with a QTL associated with NFF [20]. Isemura et al. reported co-localization of QTLs controlling pod length and seed size in azuki bean [21].

### Conservation of genomic regions associated with the four traits among related legume crops

**Figure 3:**
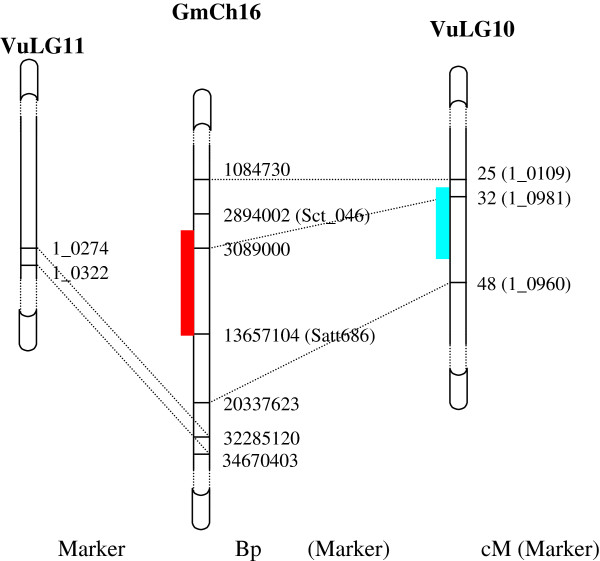
**Illustration of QTL synteny for FLT between asparagus bean and soybean.** Left: asparagus bean LG11; Middle: regional-magnified soybean chromosome 16; Right: asparagus bean LG10. The blue and red rectangles indicate the QTL intevals in asparagus bean LG10 and soybean chromosome 16, respectively. Chromosome regions that are syntenic were linked by dashed lines.

Compared to earlier mapping studies in cowpea that were based on RAPD, AFLP or solely SSR markers, the use of the genic SNPs and SSR based ‘ZZ’ genetic map compatible to the international cowpea consensus map made synteny-based comparative study between asparagus bean and related legume crops feasible. In this study, a major and a moderate QTL for FLD were mapped on asparagus bean LG 11 and 10, respectively. A Blast-N search against the soybean genome with the asparagus bean DNA markers around FLD QTLs explicitly disclosed a syntenic relationship between *Qfld.zaas.10* and the soybean flowering time QTL *QFT04* in Chr 16 (LG J) (Figure 3), which is known corresponding to cowpea/asparagus bean linkage group (designated as VuLG hereafter) 10 and VuLG 11 [6,7]. Moreover, in chromosome 1 of the cool season legume *Lotus japonicus* that is syntenic to VuLG 11 [8], two QTLs associated with flowering time were discovered [22]. Regarding PN, we revealed that VuLGs 2 and 4 each carries an associated QTL; coincidently, in the soybean chromosomes 11 and 20 that are syntenic to VuLG 2 and 4 [6,7], there have been found two QTLs for PN [23]. In common bean, QTLs associated with PN have also been mapped onto chromosome regions syntenic to VuLG 2 [24]. Taken together, the synteny of horticultural QTLs among major food legume species indicates conserved mechanisms to control these traits.

Genetic basis of NFF and LS have been rarely characterized in legumes thus far. A single large effect QTL for NFF was detected in VuLG 2 based on an AFLP-based genetic map [14]. According to [6], this linkage group corresponds to VuLG 11, where a major QTL for NFF was found. Four QTLs for NFF have been identified in garden pea [20], but unfortunately the syntenic relationship between the genomes of pea and cowpea/asparagus bean has yet to be established. Cui et al. detected seven QTLs associated with leaf senescence in soybean LG C_1_, O and D_1b+w_[25]; from the asparagus bean syntenic chromosomes (LG 7 and 11) we also found two QTLs conditioning LS. Clearly, genomic regions associated with these traits are largely conserved among related legume crops.

### Future perspectives and challenges in marker-assisted asparagus bean breeding

The identification of QTL positions in the current study has laid a preliminary foundation for maker assisted selection (MAS) for asparagus bean; however, there is still a long way to go to achieve practically efficient MAS in the field, as 1) the QTL intervals are still large; 2) although a few minor QTLs were identified for each trait, many of them showed relatively low LOD scores or were environment-dependent. Further validation of these minor QTLs are necessary before utilizing them in breeding; 3) epistatic interactions are present for certain traits; and 4) co-location of QTLs may affect pyramid of desired traits. Here, even though ZN016 carries desired QTLs conferring delayed senescence and greater pod number per plant, the effort to introgress, for instance, the major *Qls.zaas-11* would at the same time delay flowering. Thus, more precisely dissecting the linked QTLs or screening for germplasm with unlinked QTLs is necessary. Fortunately, flowering-independent control of leaf senescence has been reported in *Arabidopsis*[26]. If the same case also exists in asparagus bean, it would undoubtedly shed light on the possibility of pyramiding earliness and longevity into a single variety.

To assist narrowing QTL intervals, some recent resources available for cowpea can be used. These include a denser consensus genetic map of cowpea [6,7], a unigene data reservoir consisting of tens of thousands of transcript assemblies [6], and a genome wide association study (GWAS) panel for asparagus bean [5]. At present, a work toward verification/more precisely mapping of QTLs for pod related traits by GWAS is now underway in our lab.

## Conclusion

Introgressing favorable alleles is more easily accomplished by intervarietal crossing than by incorporating far-related resources such as wild progenitors. Therefore, dissecting genetics of horticultually important traits in an intervarietal population is not only theoretically useful, but can direct us to rapidly choose appropriate DNA markers to aid selection. The ‘ZZ’ map, which integrated genic SNPs and user-friendly SSR markers is particularly useful in this regard. The mapping of QTLs including epistatic loci for four horticulturally important traits in the current study solidified the basis for implementing marker-assisted breeding toward genetic improvement of asparagus bean.

## Methods

### Plant materials and experimental design

Two hundred and nine F_8:9_ recombinant inbred lines (RILs) produced by single seed descent from the cross of asparagus bean varieties ‘ZN016’and ‘ZJ282’ were used for collecting phenotypic data. ‘ZN016’ is a landrace asparagus bean accession originating from Southern China while ‘ZJ282’ is a commercial cultivar grown nationwide.

Four field trials were performed in 2009 and 2010 in a randomized complete block design at the normal planting season. The dates for seed sowing for each experiment were: HN2009, 16th April; SX2009, 28th April; HN2010, 28th April; SX2010, 29th April. In each year, one trial was carried out in Haining (HN, 30°32^′^N, 120°41^′^E) and the other in Shaoxing (SX, 29°43^′^N, 120°14^′^E), which are ~ 150 km apart. Except for the SX2009 trial that had no biological replicate, each experiment consisted of two replicates. For each plot ten seeds per RIL line were planted every 28 cm in 25 m-long plots on rows 75 cm apart, but only four uniform seedlings per line were retained after seedling emergence because of the big size and strong climbing habit of adult asparagus bean plants that are difficult to manage. Twelve healthy seedlings of each of the parental lines were set for each experiment. Aside each plant, a bamboo pole was erected to support plant climbing. The plots were spaced by 50 cm to avoid border effect.

### Trait evaluation

Traits evaluated include days from sowing to first flowering (FLD), nodes to first flower (NFF) on the main stem, pod number per plant (PN) and leaf senescence index (LS). FLD and NFF were both recorded on a daily basis from the beginning of flowering until the latest flowering line had flowered. PN and LS were always scored on the same day, when were 78 and 81 days after sowing in 2009 and 2010, respectively. No new pod emerged after the dates of scoring. LS was scored using a visual index ranging from 0 to 5 expressed as a percentage, where 0 was no sign of leaf senescence (complete green) and 5 equaled 100% leaf senescence (complete yellow). Indices from 1 to 4 were: 1 ≤ 20%, 2≤40%, 3≤40%, 4≤80% of leaf senescence. Data except for NFF of the 2010SX trial were collected for both replicates of all trails.

### Statistical analysis

Data from all the 209 lines were analyzed for frequency distribution using the software Statistica 6.0 (StatSoft, 2002), and for correlation and analysis of variance (ANOVA) using Data desk v.6.2 (Data Description, Ithaca, NY). Pearson’s correlation coefficients among trials were investigated using the mean phenotypic data for the two replicates of each trail. The broad-sense heritability was calculated with the formula h^2^ = *σ*_*g*_^2^/(*σ*_*g*_^2^ + *σ*_*e*_^2^), using variance components estimated based on ANOVA, where σ_g_^2^ is the genetic variance and σ_e_^2^ is the experimental error.

### QTL mapping

A subset of 96 lines of the RIL population, which had been previously genotyped by SNP and SSR markers to construct the ‘ZZ’ genetic map was used for QTL mapping [8]. Software employed for detecting QTLs was MapQTL 5.0 [27]. QTLs were first detected through interval mapping (IM), then the markers closest to the QTL peak were fixed as cofactors in multiple-QTL mode (MQM) analyses to confirm the QTLs and to scan for new QTLs. The empirically high LOD threshold of 3 was used to reliably determine major QTLs and a set of permutation-based LOD thresholds were used to try to identify possible minor QTLs. LOD thresholds were determined at the 0.05 significance level for each linkage group of each trail by 1,000 random permutations [28]. The highest LOD significance threshold for each linkage group across all experiments was used as the final LOD threshold. QTLs detected at the same chromosome location in different environments were considered as a single QTL.

### Epistasis detection

The genome wide two-locus epistasis interactions were surveyed using the ‘interactions’ function implemented in the software Map Manager QTXb20 [29]. The *P*-value cutoff was set as 1 × 10^-5^ for the total effects and 0.01 for the interaction effects of two examined loci. The statistical significance threshold for each trail was determined by 1,000 random permutations (highly significant) under the additive model with interaction.

## Competing interests

The authors declare that they have no competing interests.

## Authors’ contributions

Experiments were designed by PX and GL. XW and BW constructed the RIL population. All authors participated in field traits evaluation. PX and TH analyzed all the data and performed statistical analyses. PX drafted the manuscript and GL contributed by critical reading. All authors read and approved the final manuscript.

## Supplementary Material

Additional file 1 Across-trait correlation coefficiencies in different trials. Click here for file
